# Fluorescent Imaging for Assessment of the Effect of Combined Application of Electroporation and Rifampicin on HaCaT Cells as a New Therapeutic Approach for Psoriasis

**DOI:** 10.3390/s130303625

**Published:** 2013-03-14

**Authors:** Biliana Nikolova, Anelia Kostadinova, Borislav Dimitrov, Zhivko Zhelev, Rumiana Bakalova, Ichio Aoki, Tsuneo Saga, Iana Tsoneva

**Affiliations:** 1 Institute of Biophysics and Biomedical Engineering, Bulgarian Academy of Sciences, Acad.G. Bonchev Str., bl. 21, Sofia 1113, Bulgaria; E-Mails: nikolova@bio21.bas.bg (B.N.); anik@bio21.bas.bg (A.K.); bobi.tsvetanov@gmail.com (B.D.); zh_zhelev@yahoo.com (Z.Z.); itsoneva@bio21.bas.bg (I.T.); 2 Medical Faculty, Trakia University, 11 Armeiska Str., Stara Zagora 6000, Bulgaria; 3 Molecular Imaging Center, National Institute of Radiological Sciences, 4-9-1 Anagawa, Inage-ku, Chiba 263-8555, Japan; E-Mails: aoki@nirs.go.jp (I.A.); saga@nirs.go.jp (T.S.); 4 Medical Faculty, Sofia University, 1 Koziak Str., Sofia 1407, Bulgaria

**Keywords:** fluorescent imaging, electroporation, electrochemotherapy, rifampicin, psoriasis

## Abstract

The study aimed to clarify the role of electric pulses in combination with chemotherapy on the viability of keratinocyte cell line HaCaT, in the context of its application as a new therapeutic approach for psoriasis. The data show that electroporation of HaCaT cells in combination with rifampicin induces cytoskeleton disruption and increases permeability of cell monolayer due to cell-cell junctions' interruption, visualized by fluorescent imaging of E-cadherin and actin integrity. This was accompanied with synergistic reduction of cell viability. The study proposes a new opportunity for more effective skin treatment than chemotherapy. The future application of this electrochemotherapeutic approach for combined local treatment of psoriasis may have serous benefits because of a high possibility to avoid side-effects of conventional chemotherapy.

## 1. Introduction

One of the multiple aspects of high electric field-induced effects on the cell membrane is a transient pore formation. The process is known as electroporation. Under suitable conditions, large exogenous chemical species such as DNA, antibodies or drugs can be introduced through the pores. This technique has been developed for highly effective transfection of bacterial and eukaryotic cells [1]. Currently, the electroporation has been exploited for enhanced delivery of chemotherapeutic drugs such as bleomycin or cisplatin into tumor cells, termed “electrochemotherapy”. The increased cytotoxicity of chemotherapeutics minimizes side-effects, which is the main goal of electrochemotherapy [2–7].

Electrochemotherapy has a high antitumor effectiveness in experimental and clinical conditions and could be consider as a powerful method for treatment of several types of skin cancer or other pathological conditions affecting the skin integrity (e.g., psoriasis). The epithelial cells (keratinocytes) are the target of electrochemotherapy as a part of the skin barrier.

Psoriasis is a very common chronic skin condition, which is estimated to affect around 2% of the population (people of all ages) including men and women equally. The exact cause of psoriasis is not fully understood. The immune system is most certainly involved and appears to be overactive in the way that causes inflammation and also increased turnover of the skin cells [8]. The psoriasis treatment aims to interrupt the cycle of the increased production of skin cells. Recently, several reports for psoriasis treatment with oral rifampicin application were published [9–11]. These studies have found that 600 mg of rifampicin per day for at least 60 days can improve the symptoms [10]. However, the risk of side-effects and benefits of treatment must be considered. Rifampicin is semi-synthetic antibiotic [12], which inhibits DNA-dependent RNA-polymerase in bacterial cells, thus preventing the transcription. On the other hand, it has been shown to have effects on gene-regulatory pathways in mammalian cells, suggesting that the drug may have effect on them independently of its antimicrobial properties [9]. The role of rifampicin in the management of psoriasis is not only antibacterial in eruptive type psoriasis, but also immunosuppressive benefits were seen in several trials [10–12]. In these studies, rifampicin is well-tolerated, however the risk of hepatotoxicity cannot be ignored [8]. The main reason of the observed hepatotoxicity is due to the alteration of the integrity of hepatocytes tight junctions [13]. The reduced cell junctions respectively the reduced cell proliferation under our conditions (combination of rifampicin and electroporation) compairing with hepatocytes (rifampicin alone) could be helpful for the future local electrochemotherapy of psoriasis.

The aim of our study was to clarify *in vitro* whether electroporation can be applied as an effective trance-dermal drug delivery method for local psoriasis treatment with rifampicin, avoiding side-effects of high dose per oral administration.

Despite the wide use of electroporation many questions about the entire biophysical mechanisms are still open. Going back to the cellular level, the cytoskeleton provides the essential functions of viable cells, as shape maintaining, cell-matrix and cell-cell interactions. The application of external electric pulses can alter the cytoskeleton reorganization, thus affecting the cell adhesion. For instance, changes in the cytoskeletal structure have been observed during electroporation [14–16] and electrotransfer [17]. Actin redistribution has been reported in several studies [18,19] and in electroporation-based therapies [14,19]. Adherent junctions are formed from trans-membrane adhesive protein-cadherin (E-cadherin), localized at cell border, which could be also affected by external electric pulses application [16].

In this report, we try to clarify the effect of electric pulses alone or in combination with rifampicin on the viability of keratinocyte cell line HaCaT, e.g., alteration of cytoskeleton and actin filaments reorganization. The aim of this study is to obtain a deeper look on reversibility of the treatment and influence of the cell viability by combination of electroporation with rifampicin, using plated adherent cell line as an *in vitro* model and fluorescent imaging.

## 2. Materials and Methods

### 2.1. Chemicals

Rifampicin was purchased from Actavis (Sofia, Bulgaria). Rifampicin (MW: 823 Da) is a bacterial antibiotic of the rifamycin group. It is a semi-synthetic compound, derived from *Amycolatopsis rifamycinica*. In this study, 20 μg·mL^−1^ of rifampicin was used. According to our preliminary experiments, this amount is the minimum, indicating a cytotoxic effect on cultured cells. All other chemicals (analytical or HPLC grade) were purchased from Sigma (Steinlinz, Germany) or Lonza (Verviers, Belgium).

### 2.2. Cells

Cell line HaCaT *in vitro* spontaneously transformed keratinocytes from histologicaly normal human skin was used as a model of psoriasis. The cell line was grown as monolyer [DMEM medium high glucose, supplemented with 2 mM l-glytamine, 10% fetal calf serum (FCS), and 1% antibiotic] at 37 °C in an incubator with humid atmosphere and 5% CO_2_. Cells were passaged two times weekly by tripsinization.

### 2.3. Cell Viability Assay

The viability of HaCaT cells was determined by an MTT-test (MTT: 3-(4,5-dimethylthiazol-2-yl)-2,5-diphenyltetrazolium bromide, Applichem, Darmstadt, Germany), as described by Mosmann [20]. The MTT-test was applied after application of electric pulses on cells with or without rifapicilin treatment. To evaluate the statistical significance of the cell viability reduction, a comparison between exposed and control probes was performed by Student's t-test. P-values lower than 0.05 were considered statistically significant.

### 2.4. Electroporation Protocol

The electroporation was performed by an electroporator Chemopulse III, generating bipolar pulses, used for both *in vivo* and *in vitro* studies [3,4,6,7,15,21]. Briefly, the instrument is equipped with a large voltage control within 100–2,200 V, simplified operations, a lock against unauthorized manipulations, a battery supply, an enhanced protection against electrical hazards, an autonomy providing more than 200 electroporations with one battery charge, and a recharging time for a depleted battery of less than 10 hours [6]. The electrotreatment was done by 16 biphasic pulses, each of them 50 + 50 μs duration with 20 ms pause between both phases and pause between bipolar pulses of 880 ms. In each experiment, electrodes with interelectrode distance 1.5 cm were used. The intensity of applied electric fields was respectively: 200–133 V·cm^−1^; 500–333 V·cm^−1^; and 1,000–666 V·cm^−1^. HaCaT cells (100 μL with 1.5 × 10^5^ cells) were seeded 24 h before electroporation. Rifampicin at different concentrations was added immediately before pulse delivery. For immunofluorescent staining experiments, the cells were cultivated on cover glasses, pre-coated with fibronectin. After the electrical treatment, 900 μL DMEM, supplemented with 10% FCS, was added to each sample. The controls were treated under the same conditions, but without electric pulse application.

### 2.5. Fibronectin Coating

Fibronectin (FN) was dissolved in PBS (phosphate buffered saline: 150 mM, pH = 7.4) to 20 μg·mL^−1^. The final concentration of fibronectin was chosen to ensure a surface saturation, using protein adsorption data from the literature [22]. The adsorption procedure was performed as follow: glass cover-slips (18 × 18 mm; Assistent, Winegor, Germany) were placed in 6-well tissue culture plates (Costar, Germany) and coated with 20 μg·mL^−1^ of FN for 30 min at room temperature. Then, the plates were washed three times with PBS and 1 mL suspension of 1.5 × 10^5^ HaCaT cells was added left to spread for 24 h in humidified CO_2_ incubator. This protocol was used for study of immunofluorescent visualization of actin and E-cadherin.

### 2.6. Actin Staining

HaCaT cells with density of 1.5 × 10^5^ cells/mL were cultivated on cover glasses (18 × 18 mm), placed in 6-well plates. After 24-hour incubation, the cells were electroporated in a basal cell medium and were cultivated additionally for a period of 24 h in full cell medium. After the incubation period, the non-adhered cells were removed by triple rinsing with PBS (pH 7.4). The adhered cells were fixed with 1 mL 3% solution of paraformaldehyde (PFA) for 15 minutes at room temperature. The fixed cells were permeabilized using 1 mL 0.5% solution of Triton X-100 for 5 minutes and then incubated with 1 mL 1% solution of serum bovine albumin (BSA) for 15 minutes. The samples were washed three times with PBS (pH 7.4) and incubated for 30 min at room temperature with BODIPY 558/568 phalloidin. Again, the samples were washed three times with PBS and once with distilled water, and then were installed on objective glasses by Mowiol. The samples were analyzed using inverted fluorescent microscope (Leica DMI3000 B, Leica Microsystems GmbH, Wetzlar, Germany) with objective HCX PL FLUOTAR 63×/1.25 oil.

### 2.7. E-Cadherin Staining

For visualization of cell-cell contacts (cell adhesion contacts), HaCaT cells with density of 1.5 × 10^5^ cells/mL were cultivated on cover glasses (18 × 18 mm), placed in 6-well plates. After 24-hour incubation, the cells were electroporated in a basal cell medium and were cultivated additionally for a period of 24 h in full cell medium. After the incubation period, the non-adhered cells were removed by triple rinsing with PBS (pH 7.4). The cells were fixed with 3% PFA for 5 min, then permeabilized using 1 mL 0.5% Triton X-100 (Merck, Darmstadt, Germany) in PBS for 5 min—after saturation with 1% albumin (BSA) in PBS for 15 min. The samples were washed three times with PBS and the cells were incubated with monoclonal anti-E-cadherin antibody as a primary antibody (Dianova, Hamburg, Germany) for 30 min. Then, the samples were washed again three times with PBS and incubated for 30 min with Cy2-labelled goat anti-mouse IgG (Jackson Immunoassay Laboratories, West Grove, PA, USA) as a secondary antibody. Finally, all samples were washed three times with PBS and distilled water and mounted with Mowiol. The samples were analyzed using inverted fluorescent microscope (Leica DMI3000 B, Leica Microsystems GmbH) with objective HCX PL FLUOTAR 63×/1.25 oil.

## 3. Results and Discussion

**Figure 1.**
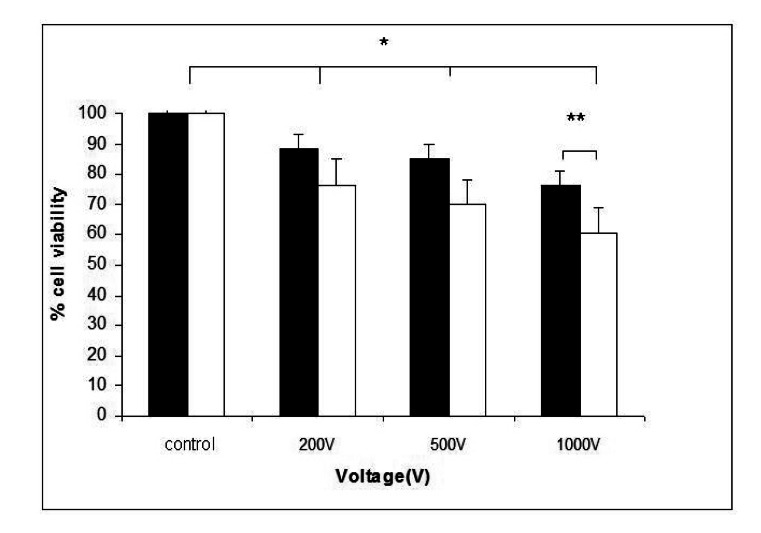
Viability of HaCaT keratinocytes after treatment with high voltage electric pulses alone (black columns) or in combination with rifampicin (white columns). Bars—SD (Standard Deviation); * p < 0.05 *versus* control groups; ** p < 0.01, significant difference between electroporated cells in the absence of rifampicin and electroporated cells in the presence of 20 μg·mL^−1^ rifampicin.

The cell viability after treatment with high voltage electric pulses alone or in combination with rifampicin was given in Figure 1. The experiments were carried out to clarify whether the electroporation in combination with rifampicin will additionally decreased the cell viability. A statistically significant reduction of cell viability was detected 24 h after pulse application. There was a negative correlation between cell viability and voltage of the applied pulses. About 20% reduction of cell viability was achieved at 1,000 V. When 20 μg·mL^−1^ of rifampicin was added before electrotreatment, an additional decrease of the cell viability was observed in all electroporated samples. This suggests a synergic effect of electroporation and rifampicin treatment on the viability of HaCaT keratinocytes.

Cell-cell contacts are essential for maintenance of the barrier function of the skin [23]. Recently, it was shown that remodeling of cell cytoskeleton and cell-cell contacts is a cell-specific process, associated with short-term and long-term effects and even with change in cell phenotype [14–16,24]. The E-cadherin (a trans-membrane adhesive protein) plays an essential role in these junctions. This is why the changes in monolyer integrity could increase the local concentration of the drug (e.g., rifampicin) in the psoriasis area.

**Figure 2.**
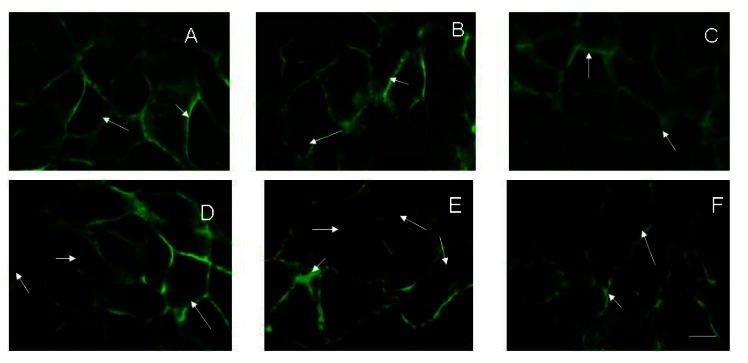
Fluorescent imaging of E-cadherin (Cy2-labelled; immunostaining) after treatment of HaCaT cells with electrical pulses and/or 20 μg·mL^−1^ rifampicin. (**A**) Untreated control HaCaT cells; (**B**) HaCaT cells, treated with 20 μg·mL^−1^ rifampicin alone; (**C**) HaCaT cells 24 h after electroporation with 1,000 V, only. (**D**–**F**) HaCaT cells treated with 20 μg·mL^−1^ rifampicin plus 200 V, 500 V, 1,000 V, respectively. Magnification—63×.

To evaluate the effect of rifampicin alone or in combination with electroporation on the stability of cell-cell contacts the confluent HaCaT cells were used. The results showed a significant loss of E-cadherin from cell junctions, which depended on the voltage applied (Figure 2). The loss of E-cadherin is associated with a permanent damage of the cell membrane and disruption of the cell monolayer. This suggests that electrotreatment could be a reason for disruption of the barrier function of the skin, reducing monolayer permeability. In Figure 2(A), the fluorescent image shows confluent HaCaT cells with well-defined cell-cell contacts and an expression of E-cadherin. After addition of 20 μg·mL^−1^ rifampicin alone to the confluent cells (Figure 2(B)), some loss of E-cadherin was detected. The application of 1,000 V electric pulse on the confluent cells also led to a loss of E-cadherin junction (Figure 2(C)). The effects of the combination of 20 μg·mL^−1^ rifampicin and electroporation at different voltages were presented in Figure 2(D–F). The images show that the cell junction integrity is compromised starting with 200 V and a synergistic effect of combined treatment is available. The effect of (1,000 V + 20 μg·mL^−1^ rifampicin) on E-cadherin distribution was irreversible and even 24 h after treatment, a diffused expression and a poor organization of the junction signal could be observed (Figure 2(F)).

**Figure 3.**
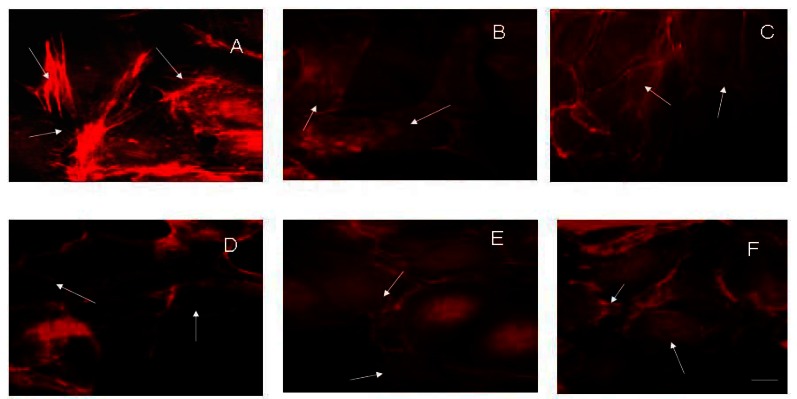
Fluorescent imaging of actin (BODIPY^558/568^ phalloidin-labelled) after treatment of HaCaT cells with electrical pulses and/or 20 μg·mL^−1^ rifampicin. (**A**) Untreated control HaCaT cells; (**B**) HaCaT cells, treated with 20 μg·mL^−1^ rifampicin alone; (**C**) HaCaT cells 24 h after electroporation with 1,000 V, only. (**D**–**F**) HaCaT cells treated with 20 μg·mL^−1^ rifampicin plus 200 V, 500 V, 1,000 V, respectively. Magnification—63×.

It is well known that actin plays an important role in maintaining of cell junctions. It was logical to expect changes in integrity of the actin cytoskeleton after treatment with electrical pulses and rifampicin. The effects of electroporation and/or rifampicin on HaCaT actin cytoskeleton are presented in Figure 3. Before electroporation, the cells displayed intact actin filament with a lot of stress fibers and spindle shape (Figure 3(A)). After the treatment of keratinocytes with 20 μg·mL^−1^ rifampicin, some changes in actin filaments could be observed. The stress fibers were less expressed and the shape of the cells was altered. Diffused organization of actin filaments can be recognized at Figure 3(C) as a result of electric field application of 1,000 V on the cell monolayer. Rifampicin in combination with electrical pulses significantly modified the shape of HaCaT cells. The influence of electroporation on the actin cytoskeleton was dramatic. Even at (200 V + 20 μg·mL^−1^ rifampicin) the number of stress fibers was diminished and the cells were not spread very well (Figure 3(D)). At 1,000 V + 20 μg·mL^−1^ rifampicin the stress fibers totally disappeared and the cells become rounded (Figure 3(F)). The data from imaging analyses were in a very good correlation with cell viability. Such alteration of actin cytoskeleton *in vivo* could lead to disturbance in the integrity of the tissue and eventually its complete maceration.

Rifampicin is a poorly adsorbed antibiotic that have been effectively used for treatment of several bacterial deceases [12]. Psoriasis is one of the more difficult skin conditions to treat. The aim of psoriasis treatment is to interrupt the cycle that causes an increased production of skin cells.

Our study shows that electroporation alone or in combination with rifampicin influences the viability and adherent properties of keratinocytes *in vitro*. A significant synergistic reduction of cell viability was observed in case of combined application of high voltage electrotreatment (1,000 V) and 20 μg·mL^−1^ rifampicin (Figure 1). This was accompanied with actin filaments reorganization and loss of E-cadherin from cell-to-cell junctions, which was visualized by fluorescent imaging (Figures 2 and 3).

The cytoskeleton changes, induced by electroporation, are known to disrupt epithelial barrier function, which could be assumed as a reason for increase of the epithelial monolayer permeability [15,25]. We established that the dissociation of actin filaments, as well as the distribution of adherent junctions correlated with increasing of voltage (200–1,000 V) of electric pulses in the presence of rifampicin in concentration 20 μg·mL^−1^. These results are in agreement with reports in the literature, demonstrating that cytoskeleton and morphological changes in epithelial cells influence cell viability [15,16].

An interesting observation is that the changes in cytoskeleton reorganization are stronger in comparison with the proliferation activity, measured by MTT assay. Xiao *et al.* [26] have shown that electrical pulses reduced number of apoptotic cells. Our data are in agreement with this observation, due to the fact that MTT test measured the mitochondrial activity, respectively it is connected with mitochondrial trans-membrane potential and apoptotic pathway. However, the molecular reason for the changes in this signaling mechanism is not clear yet and need further investigation.

## 4. Conclusions

The described data show that electroporation of HaCaT cells in combination with rifampicin induces cytoskeleton disruption and increases permeability of cell monolayer due to cell-cell junctions' interruption, followed by reduction of cell proliferation. The study proposes a new opportunity for more effective skin treatment than chemotherapy. The future application of this electrochemo-therapeutic approach for combined local treatment of psoriasis may have serous benefits because of a high possibility to avoid side-effects of conventional psoriasis chemotherapy.
